# The First Shared Online Curriculum Resources for Veterinary Undergraduate Learning and Teaching in Animal Welfare and Ethics in Australia and New Zealand

**DOI:** 10.3390/ani5020362

**Published:** 2015-05-29

**Authors:** Jane Johnson, Teresa Collins, Christopher Degeling, Anne Fawcett, Andrew D. Fisher, Rafael Freire, Susan J. Hazel, Jennifer Hood, Janice Lloyd, Clive J. C. Phillips, Kevin Stafford, Vicky Tzioumis, Paul D. McGreevy

**Affiliations:** 1 Faculty of Veterinary Science, University of Sydney, Sydney, NSW 2006, Australia; E-Mails: anne.fawcett@sydney.edu.au (A.F.); jennifer.hood@sydney.edu.au (J.H.); vicky.tzioumis@sydney.edu.au (V.T.); paul.mcgreevy@sydney.edu.au (P.D.M.); 2 School of Veterinary and Life Science, Murdoch University, Murdoch, WA 6150, Australia; E-Mail: t.collins@murdoch.edu.au; 3 Sydney Medical School, University of Sydney, Sydney, NSW 2006, Australia; E-Mail: chris.degeling@sydney.edu.au; 4 Faculty of Veterinary Science, University of Melbourne, Parkville, VIC 3010, Australia; E-Mail: adfisher@unimelb.edu.au; 5 School of Animal and Veterinary Science, Charles Sturt University, Sutherland Laboratories, Wagga Wagga, NSW 2650, Australia; E-Mail: rfreire@csu.edu.au; 6 School of Animal and Veterinary Science, University of Adelaide, Roseworthy, SA 5005, Australia; E-Mail: susan.hazel@adelaide.edu.au; 7 College of Public Health, Medical and Veterinary Science, James Cook University, Townsville, QLD 4811, Australia; E-Mail: janice.lloyd@jcu.edu.au; 8 School of Veterinary Science, University of Queensland, Gatton, QLD 4343, Australia; E-Mail: c.phillips@uq.edu.au; 9 Institute of Veterinary, Animal and Biomedical Science, Massey University, Palmerston North 4442, New Zealand; E-Mail: k.j.stafford@massey.ac.nz

**Keywords:** animal ethics, animal welfare, online curriculum resources, learning and teaching, scenarios, quality of life assessment

## Abstract

**Simple Summary:**

There is a need for teaching Animal Welfare and Ethics in veterinary schools and we are developing online resources to meet this need. In this paper we describe how we prioritized the development of these resources by polling experts in the field.

**Abstract:**

The need for undergraduate teaching of Animal Welfare and Ethics (AWE) in Australian and New Zealand veterinary courses reflects increasing community concerns and expectations about AWE; global pressures regarding food security and sustainability; the demands of veterinary accreditation; and fears that, unless students encounter AWE as part of their formal education, as veterinarians they will be relatively unaware of the discipline of animal welfare science. To address this need we are developing online resources to ensure Australian and New Zealand veterinary graduates have the knowledge, and the research, communication and critical reasoning skills, to fulfill the AWE role demanded of them by contemporary society. To prioritize development of these resources we assembled leaders in the field of AWE education from the eight veterinary schools in Australia and New Zealand and used modified deliberative polling. This paper describes the role of the poll in developing the first shared online curriculum resource for veterinary undergraduate learning and teaching in AWE in Australia and New Zealand. The learning and teaching strategies that ranked highest in the exercise were: scenario-based learning; a quality of animal life assessment tool; the so-called ‘Human Continuum’ discussion platform; and a negotiated curriculum.

## 1. Introduction

Australian and New Zealand community concerns and expectations about Animal Welfare and Ethics (AWE) have risen steeply in the last decade, and continue to grow [1]. Veterinary graduates are in unique position to lead in this area, both conceptually and practically, including through current debates and regulatory processes [2]. Increasingly, veterinary accreditation is also recognizing the need to incorporate AWE in undergraduate courses. Indeed, the World Organisation for Animal Health (OIE) now recommends that Day One veterinary graduates “should be the leading advocates for the welfare of all animals, recognizing the key contribution that animals make to human society through food production, companionship, biomedical research and education” [3]. Further, it is suggested that an increased awareness of welfare science and ethics through formal education should raise the standards of veterinary care [3,4]. This paper describes part of an ongoing project that aims to ensure Australian and New Zealand veterinary graduates develop the knowledge, and the research, communication and critical reasoning skills, to fulfill the AWE role expected of them in contemporary society. Specifically, this paper articulates the role of a modified deliberative poll in developing the first shared online curriculum resources for veterinary graduate learning and teaching of AWE in all Australian and New Zealand veterinary schools.

### 1.1. The Importance of AWE for Veterinarians

AWE is important to veterinarians for a number of reasons. In the first instance, public concern around this issue is increasing [1,5]. In the Australian context, there has been renewed unease over live animal export and worries over foreign methods of slaughtering Australian livestock [6]. Meanwhile, in the Organisation for Economic Co-operation and Development (OECD) nations, growing public agitation about AWE has led to reviews of animal welfare standards and guidelines [7,8].

Second, the global push towards food security and sustainability demands veterinary graduates be leaders in, and ambassadors for, animal welfare and ethical food production. In Australia and New Zealand veterinarians have an increasing role in South East Asia in areas of animal health and management (e.g., via Australian aid programs, such as AusAid), so appropriate training of graduates will have a significant impact on the entire region. This is especially important given the relationship between animal welfare and susceptibility to disease, and that a global outbreak of disease is likely to occur in South East Asia [9].

Third, veterinarians are professionals who work in direct contact with animals and have obligations which follow from this as well as expectations associated with their role. As the Report on European Veterinary Education in Animal Welfare states, “veterinarians have a professional and ethical responsibility to use their scientific knowledge and skills for the benefit of animal welfare” [10]. This entails obligations to a range of parties including clients, patients, colleagues, the community, society, and themselves. Navigating these sometimes conflicting obligations demands a detailed understanding of AWE and practice in ethical decision making [11]. For instance, veterinarians play vital roles in regulating the care and use of animals in areas where human and animal needs are in tension such as in research institutions when veterinarians act as animal welfare officers or members of Animal Ethics Committees. Production animal veterinarians face similar challenges with food animal industry bodies required to meet societal expectations around animal welfare and improve transparency about husbandry practices and slaughter [12,13].

Finally, an understanding of AWE is increasingly likely to be mandated through accreditation of undergraduate veterinary programs with the Royal College of Veterinary Surgeons (RCVS) Day One competences already demanding animal welfare related skills [14].

In spite of its importance to veterinary practice, there is room for improvement in how AWE is taught. Undergraduate veterinary education typically focuses on animals and their welfare to develop veterinary professionals as scientific advocates for animals in many contexts, but sometimes includes only brief teaching of professional, animal and veterinary ethics. There is a compelling argument, therefore, for ensuring core competencies in AWE are part of veterinary undergraduate, and indeed postgraduate, education.

### 1.2. What Skills Are Required?

A suite of skills is required to properly prepare graduates in AWE. These include knowledge of animal welfare science, proficiency in critical thinking and ethical reasoning, and competency in communication.

To be effective in meeting societal expectations about proficiency in assessing welfare issues and making treatment recommendations [15], veterinary graduates need to know how to identify what constitutes good and bad animal welfare for different animal species and for different categories of animal (e.g., companion, livestock, wild) and how to provide advice on species found in different contexts (e.g., rabbits considered as companions, research subjects or feral). It is important for veterinary graduates to be able to use objective and subjective measures of welfare, and implement strategies to achieve good welfare for animals.

Skills in ethical reasoning are needed as ethical dilemmas are common in veterinary practice. For example, 57% of veterinarians surveyed in the UK reported one to two stressful ethical dilemmas a week, and 34% reported three to five dilemmas a week [16]. Graduates commented that the level of undergraduate training in ethics was inadequate, and that high levels of stress were associated with ethical dilemmas, which might have been due to lack of knowledge about how to process and deal with such dilemmas [16]. Veterinary students may not progress in their moral reasoning during their undergraduate years, as research shows that similar ethical reasoning is used by students from the first to the final year [17]. This should be addressed since there is evidence that higher levels of moral reasoning are associated with improved clinical practice [18]. According to May, “in parallel with classes aimed at sequential development of clinical reasoning it is important to introduce students to and help them develop systems that aid the resolution of ethical dilemmas” [19]. This involves learning how to analyze dilemmas, identifying and evaluating the relevant issues, understanding the values at play (including their own) and reaching an ethically defensible conclusion based on scientific, economic and other forms of evidence, rather than on peer or parent attitudes, assumptions, intuitions or emotions. In developing these skills, students will appreciate that ethical dilemmas are vexed, probably inevitable in their profession and frequently have no clear resolution.

The communication skills required to fulfill the AWE role demanded of contemporary veterinarians include competence in explaining their ethical reasoning. Animal welfare involves both value-based and scientifically-based decisions [20], and veterinarians must be able to communicate with people who have differing views of animal welfare. Finally, veterinarians can, since they are held in high esteem by the public, lead discussions on policy development, educate the public on contemporary issues (e.g., on responsible pet ownership) and provide an informed and reasoned voice on complex and often emotional issues (e.g., intensive farming systems).

### 1.3. How Can These Skills Be Best Developed in Veterinary Students?

To ensure that the next generation of veterinarians in Australia and New Zealand are scholarly, judicious and well-informed in AWE, a team comprising representatives from the veterinary schools in these countries was assembled and an Australian Office of Learning and Teaching (OLT) grant secured. The OLT project began in early 2014 with the aim of developing new shared curriculum resources for AWE in Australia and New Zealand, linked to an online portal.

#### The Online Portal

Higher education is increasingly moving online. Building an online portal as part of this project aligns with this trend and with AWE projects being undertaken at other veterinary tertiary institutions (e.g., Michigan State University [21], The University of Edinburgh and Scotland's Rural College [22]) and other animal welfare charities and non-governmental organizations, such as World Animal Protection (formerly the World Society for Protection of Animals) [23].

An online portal has numerous advantages that make it an ideal mechanism to foster development of the AWE skills outlined above. For instance, it can furnish a single forum for students, academics and graduates and ensure consistency in the material delivered across a range of institutions. An online portal also gives flexibility to these institutions in how they use shared material, allowing them to access resources as appropriate, and to tailor content to the particular needs of students and to balance an institutional context or focus (e.g., on production animals *versus* companion animals). With effective management, resources can be kept updated to ensure relevance. Finally, an online portal can facilitate fruitful interactions between students at different institutions, honing their communication skills and their understanding of different ethical viewpoints, as well as allowing the pool of AWE expertise in Australia and New Zealand to be shared.

## 2. Methods

To determine the learning activities best able to achieve the AWE skills and learning outcomes identified as crucial to veterinary graduates, we engaged in a modified form of deliberative polling with an invited panel of leaders (n = 8) in the field of animal welfare education in Australia and New Zealand. As its name suggests, deliberative polling exercises incorporate deliberative methods into traditional polling [24]. Typically, a group of people are given information and opportunities to discuss a topic and then take a vote. As with traditional polls, the outcomes produced from a deliberative poll are aggregated individual opinions (rather than consensus), but the opinions are shaped by the prior group deliberation.  The exercise was spread over a two-day workshop funded by the OLT and held at the Faculty of Veterinary Science at the University of Sydney, Australia. On the first day the disciplinary leaders were provided with information regarding eight different teaching tools and learning activities. The tools/activities were described to the panel as follows:

### 2.1. A Negotiated Curriculum

A system that allows cohorts of students to vote for the AWE topics they wish to focus on each semester of a veterinary degree course. There is an extensive literature related to the negotiation of learning contracts by individual course members and examples of group negotiation processes [25,26]. An online portal is an ideal mechanism to facilitate this form of negotiated curriculum as it can enable students and teachers to easily link to the pool of resources related to each topic and tailor the curriculum to their particular needs. To deliver tools for learning outcomes that address all AWE competences, a wide range of learning scenarios would be developed so that cohorts of students could select those that best align with their experience and background. Enabling students to vote on the topics to be studied each semester empowers and engages students, allowing them to focus on issues of interest and relevance and to take responsibility for the development of their own ethical frameworks.

### 2.2. Scenarios for Case-Based Learning

After completing initial modules in both ethical theory and animal welfare science, students would encounter a series of scenarios that demand application of an ethical framework. These scenarios would be the primary means to introduce online materials to students. The scenarios would cover an array of veterinary contexts from simulated clinical cases to questions of professional conduct. Scenarios offer students exposure to the ethical and welfare issues they will face in practice in a safe learning environment where they can try out and refine alternative approaches, undertake other parts of the scenario where appropriate, see how others have handled a given situation, make mistakes with no untoward consequences to any stakeholder (including patients, clients, or a business/practice), correct problems, and expand their repertoire of judgment and interpersonal communication skills.

### 2.3. Automated Search Engine for Hot Topics

An automated search engine would ensure relevant and up-to-date material is generated and presented every time a new cohort of students enrolls. Providing authentic and topical resources helps ensure student engagement and can scaffold the development of ethical skills related to contemporary debates in AWE.

### 2.4. The “Human Continuum”

The “Human Continuum” is an established cooperative/cognitive learning strategy, which requires students to identify and commit to a position in response to a stimulus question. They occupy this position on a physical line marked out in the classroom. Students debate and negotiate with others on the line, listen to alternative positions and reconsider their view. The exercise stimulates thinking and learning from peers. This project would develop this tool as an online resource to help students develop argument and critical assessment skills on polarizing topics and could be used to promote engagement by participating schools. The tool can be used as an introduction to illustrate the wide range of views and the complexity and difficulty of making decisions on issues, and to help students to express justifications for their positions using ethical frameworks and principles.

### 2.5. A Quality of Life Assessment Tool (QOL)

Primarily as a teaching tool, we would develop an online calculator to bring together welfare scales from various domains that capture an animal’s quality of life. For instance, students could be asked to consider the issues that arise from the deliberate selection for exaggerated physical traits, or because of inherited disease in companion animals. Asking students to estimate the welfare impact of each disorder is innovative. Estimations would be informed by the use of a conceptual Breed-Disorder Welfare Impact Score for dogs [27].

### 2.6. Communication Training Spaces

Providing welfare advice that is scientifically-supported, clear and unbiased to clients, the public and legislators is important and expected in contemporary veterinary practice. Students can develop the knowledge and communication skills to attain this competence through online personal learning spaces. These spaces enable students to present and share their knowledge and views, and to appreciate the positions held by various stakeholders. The kind of exchange and interaction facilitated by this tool allows for the development of a rich learning environment. Maintaining these spaces throughout the course would allow students to track their progress and enhance reflective learning and practice.

### 2.7. Team Based Learning (TBL)

TBL can be used to develop communication and teamwork skills as well as to reinforce course content [28]. TBL involves students reading course material prior to ‘class’ (whether a physical or a virtual class), then answering multiple choice questions related to that material, first as an individual and then as a team, to help identify misunderstandings and gaps in knowledge. Class time is then devoted to teams applying knowledge to authentic problems. For this reason it is ideally suited to use with scenario-based learning.

### 2.8. Personal Reflection Tools

Online reflection tools can be used to allow students to assess their own attitudes to animals on entry to the portal and at the end of every year of their interaction with the portal. Members of the expert panel have developed online questionnaires to investigate whether Australian veterinary students’ attitudes to animals correlate with those reported in the international literature, and to further explore the impact of demographic and experiential factors on attitudes towards animals and career aspirations. A modified version of these questionnaires could be deployed as a reflection tool for current veterinary students.

The eight experts on the panel were given three hours to discuss, debate and reflect upon the information above. The panel reconvened the next day when they were polled using an anonymous ballot. As part of this task they were asked to rank the eight different tools in terms of their importance for ensuring Day One competences for Australian and New Zealand veterinary graduates in AWE.

## 3. Results and Discussion

### 3.1. Results

By far, the highest ranked tool was Scenarios for Case-based learning, with the QOL Assessment Tool ranked second and the Human Continuum and A Negotiated Curriculum sharing the third ranking (See Table 1). Given the weighting assigned by our assembled experts, the four resources outlined above will be a focus of the portal. The results of the panel discussion regarding the four top ranked resources are described in the Discussion below.

**Table 1:** Ranking curriculum features from 1–10; where 1 = most important and 10 = least important.

Curriculum Feature	Ranking allocated by each respondent								Ranking Median	Ranking order
	Resp. 1	Resp. 2	Resp. 3	Resp. 4	Resp. 5	Resp. 6	Resp. 7	Resp. 8		
A negotiated curriculum	2	10	3	4	5	7	10	3	4.5	3
Scenarios and learning exercises	4	1	1	1	1	1	1	2	1	1
Automated search engines for hot topics	3	7	8	3	9	6	5	9	6.5	7
A post-graduate suite	9	9	10	10	7	4	9	10	9	10
Animal welfare science essays	10	8	9	6	10	9	6	8	8.5	9
Dialogue Development tool (*i.e.*, Human Continuum)	5	3	6	7	4	5	2	4	4.5	3
Quality of Life assessment tool	1	6	2	2	2	10	4	6	3	2
Delivering welfare advice tool	8	5	4	5	8	2	3	7	5	5
Team based learning (TBL)	7	2	7	8	6	3	7	1	6.5	7
Personal reflection tools	6	4	5	9	3	8	8	5	5.5	6

### 3.2. Discussion

Drawing on the learning resources described above, the panel agreed that the project should focus on the goals articulated under the former Australian Animal Welfare Strategy (AAWS), namely: (i) a national approach to animal welfare (AW); (ii) sustainable improvements to AW; and (iii) improved communication, education and training [29]. Animals will be studied in six contexts: livestock/production animals; animals used for work, sport, recreation or display; companion animals; animals in the wild; aquatic animals; and animals used in research and for teaching purposes. These six contexts align with the AAWS framework that emerged with a goal of developing new, nationally consistent policies and enhancing extant animal welfare arrangements. Within each context, animal welfare science, veterinary clinical ethics and animal ethics, and ethical models will be considered. Because all Australia and New Zealand veterinary schools currently have accreditation from the UK’s Royal College of Veterinary Surgeons (RCVS), the panel agreed that the RCVS Day One Competencies (n = 8) relating to AWE must also be addressed.

### 3.3. Scenarios

All members of the panel were highly enthusiastic about and have experience in the use of scenarios for teaching AWE; the advantages of case-based learning, including for enhancing knowledge retention and to apply learned material, are widely recognised [30,31]. Scenarios are already used extensively in AWE teaching worldwide and have been used successfully in teaching medical ethics [32]. Well-developed scenarios are relevant and engaging for students, allowing them to acquire knowledge and skills through practical application in a safe and supported learning environment.

### 3.4. The QOL Tool

Discussions revealed the panel considered this tool could serve a number of functions. In the first instance it could be designed to help students appreciate the many elements that affect QOL in a particular context, prompting them to ask deeper questions regarding the conditions which frame the welfare of an individual animal. For instance, by considering the case of a particular animal (e.g., a bulldog with breathing difficulties), students may be required to reflect on the welfare and ethical implications of breeding practices, which promote selection for exaggerated physical traits or perpetuate inherited disease. In addition, this tool will promote discussion about the role of the veterinarian in resolving some complex issues (e.g., breeds requiring high rates of caesarean sections). In the case of companion animals, the QOL tool will help students determine what owners perceive to be of value to their animals (e.g., long walks, sleeping in the sun, playing with their owner). These values and preferred activities contribute to the animal’s QOL and need to be factored into assessing animal welfare. Discussions showed the panel also regarded this tool as a valuable resource for practising veterinarians in discussions with owners about quality of life and euthanasia.

### 3.5. The Human Continuum

The panel was excited by the possibility of developing this resource online. Online delivery will facilitate discussion and interaction across schools and avoid face-to-face peer pressure, helping students learn to take an ethical stance and to justify this, to make and refine arguments in response to challenges by their peers from a diversity of backgrounds and to listen to and assess the values and positions of others.

### 3.6. A Negotiated Curriculum

In discussions the *Negotiated Curriculum* was seen to be central to the success of the current collaboration between schools. We recognize that the collaborating schools have different fortes (e.g., production *versus* companion animals) and thus represent a diversity of views. We see this as a core strength in the anticipated development of communication tools in AWE. A crucial issue for the project is ensuring adequate and inclusive coverage of issues required to meet both accreditation standards and the requirements of formal university qualifications on the one hand, while on the other, ensuring the curriculum content is relevant and flexible to meet course members’ specific needs and context. Our response to managing this tension has been to employ a negotiated curriculum.

## 4. Conclusions

AWE is of increasing importance in veterinary education. A greater focus on Day One Competencies for veterinary graduates and emerging AWE issues, results in a need for curriculum renewal. The online portal we have begun to develop reflects the needs of veterinary students as perceived by the region’s current leaders in AWE education. It will enable state-of-the art teaching of AWE, with a focus on scenarios, a quality of life tool, an online version of the Human Continuum and a negotiated curriculum. The portal will enable consistent curriculum delivery across all eight veterinary schools in Australia and New Zealand and will facilitate a rich and engaging environment for teaching and learning. The resources we develop will not only enhance veterinary education but have the potential to be extended to disciplines such as agriculture, animal science and animal care, facilitating high quality AWE teaching throughout the Higher Education sector.

## References

[1] McGreevy P.D., Dixon R.J. (2005). Teaching of animal welfare at the University of Sydney. J. Vet. Med. Educ..

[2] Sherman D., Dennison T. Animal welfare: The importance of veterinary legislation and the role of the veterinary profession. Proceedings of the 3rd OIE Global Conference on Animal Welfare.

[3] (2012). OIE Recommendations on Competencies of Graduating Veterinarians (‘Day 1 Graduates’) to Assure National Veterinary Services of Quality.

[4] Broom D.M. (2005). Animal welfare education: Development and prospects. J. Vet. Med. Educ..

[5] Stafford K.J. (2013). Animal Welfare in New Zealand.

[6] Tiplady C., Walsh D.B., Phillips C.J.C. (2012). Cruelty to Australian cattle in Indonesian abattoirs—How the public responded to media coverage. J. Agric. Environ. Ethics.

[7] Broom D.M. (2011). A history of animal welfare science. Acta Biotheor..

[8] Webster J. (2008). Animal Welfare: Limping Towards Eden.

[9] Mackenzie J.S., Williams D.T. (2009). The zoonotic flaviviruses of southern, south-eastern and eastern Asia, and Australiasia: The potential for emergent viruses. Zoonoses Public Health.

[10] (2013). FVE & EAEVE Report on European Veterinary Education in Animal Welfare Science, Ethics and Law. http://www.fve.org/uploads/publications/docs/executive_summary_aw_day_one_competences_adopted.pdf.

[11] Rollin B.E. (2006). An Introduction to Veterinary Medical Ethics.

[12] Mellor D.J., Webster J.R. (2014). Development of animal welfare understanding drives change in minimum welfare standards. Revue scientifique et technique-office international des epizooties.

[13] Mellor D.J., Stafford K.J. (2001). Integrating practical, regulatory and ethical strategies for enhancing farm animal welfare. Austr. Vet. J..

[14] RCVS RCVS Day One Competences, 2014. http://www.rcvs.org.uk/document-library/rcvs-day-one-competences/.

[15] Yeates J.W., Main D.C. (2011). Veterinary surgeons opinions on dog welfare issues. J. Small Anim. Pract..

[16] Batchelor C.E.M., McKeegan D.E.F. (2012). Survey of the frequency and perceived stressfulness of ethical dilemmas encountered in UK veterinary practice. Vet. Rec..

[17] Quinn C., Kinnison T., May S.A. (2012). Care and justice orientations to moral decision-making in veterinary students. Vet. Rec..

[18] Bebeau M.J. (2002). The defining issues test and the four component model: contributions to professional education. J. Moral Educ..

[19] May S. (2013). Clinical reasoning and case-based decision making: The fundamental challenge to veterinary educators. J. Vet. Med. Educ..

[20] Fraser D. (2008). Understanding animal welfare. Acta Vet. Scand.

[21] Siegford J.M., Zanella A.J., Bernardo T., Heleski C.R., Wickens C.L., Laughlin K., Malinowski R. (2007). Leveraging expertise in animal welfare to create educational equity. Anim. Welf..

[22] Animal Behaviour and Welfare Course. www.coursera.org/course/animal.

[23] Coombs E. (2013). A toolbox for animal welfare education. ATLA.

[24] Fishkin J.S. Deliberative Polling: Executive Summary. http://cdd.stanford.edu/polls/docs/summary/.

[25] Boud D. (1988). Developing Student Autonomy in Learning.

[26] Brew A., Barrie S. (1999). Academic development through a negotiated curriculum. Int. J. Acad. Dev..

[27] Collins L.M., Asher L., Summers J., McGreevy P. (2011). Getting priorities straight: risk assessment and decision-making in the improvement of inherited disorders in pedigree dogs. Vet. J..

[28] Hazel S.J., Heberle N., McEwen M.-M., Adams K. (2013). Team-Based Learning (TBL) increases active engagement and enhances development of teamwork and communication skills in a first year course for veterinary and animal science undergraduates. J. Vet. Med. Educ..

[29] Australian Animal Welfare Strategy. http://www.australiananimalwelfare.com.au/content/about-aaws.

[30] Siegford J.M., Zanella A.J., Bernardo T., Heleski C.R., Wickens C.L., Laughlin K., Malinowski R. (2007). Leveraging expertise in animal welfare to create educational equity. Anim. Welf..

[31] Grauer G.F., Forrester S.D., Shuman C., Sanderson M.W. (2008). Comparison of student performance after lecture-based and case-based/problem-based teaching in a large group. J. Vet. Med. Educ..

[32] Braunack-Mayer A.J., Gillam L.H., Vance E.F., Gillett G., Kerridge I., McPhee J., Saul P., Smith D., Wellsmore H., Koczwara B. (2001). An ethics core curriculum for Australasian medical schools. Med. J. Aust..

